# When Bad Guys Become Good Ones: The Key Role of Reactive Oxygen Species and Nitric Oxide in the Plant Responses to Abiotic Stress

**DOI:** 10.3389/fpls.2016.00471

**Published:** 2016-04-12

**Authors:** Fernanda S. Farnese, Paulo E. Menezes-Silva, Grasielle S. Gusman, Juraci A. Oliveira

**Affiliations:** ^1^Laboratory of Plant Ecophysiology, Instituto Federal Goiano – Campus Rio VerdeGoiás, Brazil; ^2^Laboratory of Plant Chemistry, Univiçosa – Faculdade de Ciências Biológicas e da SaúdeViçosa, Brazil; ^3^Department of General Biology, Universidade Federal de ViçosaViçosa, Brazil

**Keywords:** crosstalk, signaling, systemic acquired acclimation, *S*-nitrosylation, *S*-glutathionylation, gene expression

## Abstract

The natural environment of plants is composed of a complex set of abiotic stresses and their ability to respond to these stresses is highly flexible and finely balanced through the interaction between signaling molecules. In this review, we highlight the integrated action between reactive oxygen species (ROS) and reactive nitrogen species (RNS), particularly nitric oxide (NO), involved in the acclimation to different abiotic stresses. Under stressful conditions, the biosynthesis transport and the metabolism of ROS and NO influence plant response mechanisms. The enzymes involved in ROS and NO synthesis and scavenging can be found in different cells compartments and their temporal and spatial locations are determinant for signaling mechanisms. Both ROS and NO are involved in long distances signaling (ROS wave and GSNO transport), promoting an acquired systemic acclimation to abiotic stresses. The mechanisms of abiotic stresses response triggered by ROS and NO involve some general steps, as the enhancement of antioxidant systems, but also stress-specific mechanisms, according to the stress type (drought, hypoxia, heavy metals, etc.), and demand the interaction with other signaling molecules, such as MAPK, plant hormones, and calcium. The transduction of ROS and NO bioactivity involves post-translational modifications of proteins, particularly *S*-glutathionylation for ROS, and *S*-nitrosylation for NO. These changes may alter the activity, stability, and interaction with other molecules or subcellular location of proteins, changing the entire cell dynamics and contributing to the maintenance of homeostasis. However, despite the recent advances about the roles of ROS and NO in signaling cascades, many challenges remain, and future studies focusing on the signaling of these molecules *in planta* are still necessary.

## Introduction

A typical plant cell has more than 30,000 genes and a large number of proteins, many of which are still unknown, and these proteins may have their activity and/or function altered by several types of post-translational modifications (Cramer et al., 2011). Therefore, at the cellular level, plant responses to the environment are extremely complex and involve interactions and crosstalk with many molecular pathways. One of the first plant responses to the environment involves reactive oxygen species (ROS) and reactive nitrogen species (RNS), which are key signaling molecules and regulate many different plant processes through the activation of secondary messengers, the induction of gene transcription and changes in enzyme activity (Gaupels et al., 2011; Mengel et al., 2013; Lamotte et al., 2015).

Reactive oxygen species is a generic term used to describe chemical species formed from the incomplete reduction of molecular oxygen. The best-known ROS include superoxide anion ($O_{2}^{•-}$), hydrogen peroxide (H_2_O_2_) and the hydroxyl radical (OH^-^; **Table 1**). ROS have distinct biological properties, a short half-life and high chemical reactivity (OH^-^ has indiscriminate reactivity to biological molecules, while $O_{2}^{•-}$ and H_2_O_2_ have preferred biological targets; del Río, 2015). Similarly, RNS is a term used to collectively refer to nitric oxide (NO) and the molecules derived from this radical (**Table 1**) (Patel et al., 1999; Rahman et al., 2012). NO is a gaseous, small, reactive molecule that readily diffuses across the cells and interacts with different cellular compounds, including other radicals (Correa-Aragunde et al., 2015). Due to their high reactivity and potential to damage cellular structures under conditions of redox imbalance, the generation of ROS and RNS in cells was originally considered to be a uniquely harmful and damaging process (Demidchik, 2015; Lushchak, 2015). Currently, however, it is known that these molecules are important components of signaling networks in various plant processes, which is possible due to the development of effective antioxidant systems that are capable, in most cases, of containing the toxicity of ROS and RNS, allowing these molecules to act as efficient signal transducers (del Río et al., 2006; del Río, 2015).

**Table 1:** Main reactive oxygen species (ROS) and reactive nitrogen species (RNS) found in plant cells (adapted from Rahman et al., 2012).

Free radicals	Non-radicals
**Reactive oxygen species**
Superoxide, $O_{2}^{•-}$	Hydrogen peroxide, H_2_O_2_
Alkoxyl, RO^•^	Hypochlorous acid, HOCl
Hydroxyl, OH^-^	Ozone, O_3_
Peroxyl, ROO^•^	Peroxynitrite, ONOO^-^
Hydroperoxyl, HO_2_^•^	Singlet oxygen, ^1^O_2_
**Reactive nitrogen species**
Nitric oxide, NO^•^	Nitrous acid, HNO_2_
Nitric dioxide, NO_2_^•^	Nitrosonium cation, NO^+^
Nitrate radical, NO_3_^•^	Nitrosyl anion, NO^-^
	Peroxynitrite, ONOO^-^
	Alkylperoxynitrites: ROONO
	Dinitrogen trioxide, N_2_O_3_

Nitric oxide and ROS are involved in and interact with each other in a wide range of cellular processes, which include response to abiotic stresses (Joudoi et al., 2013), defense against pathogens (Asai et al., 2008) and normal growth and development processes, such as germination and flowering (El-Maarouf-Bouteau and Bailly, 2008). It is easy to see, therefore, that the changes triggered by these signaling molecules are highly variable according to the environmental context. Due to the high complexity of this process, there is still much that is unclear about the signaling mechanisms triggered by ROS and NO, the interaction of these molecules with each other and with other components of the signaling pathway, and the balance between production and elimination of reactive species by antioxidants. A growing number of studies have sought to answer these questions, and many advances have been made in the field. Thus, considering the central role of these molecules in the response and adaptation of plants to changes in the environment, the present review aims to summarize the existing knowledge of the interactions between ROS and NO in the plant response to abiotic stress, focusing on the sources and production sites of these molecules, interactions with other signaling components and molecular aspects.

## Biosynthesis, Transport, and Metabolism of ROS and NO

**FIGURE 1:**
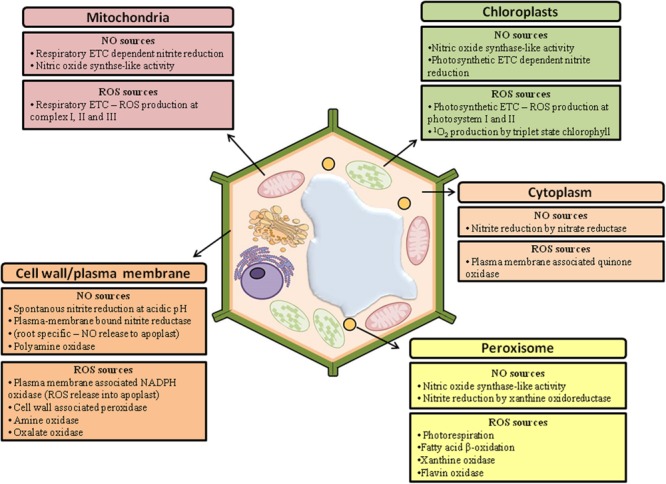
**Main sources of nitric oxide (NO) and reactive oxygen species (ROS) in plant cells (adapted from Groß et al., 2013)**.

During cell signaling in response to stress, the redox state of the plant cells is rapidly altered by both the increase in ROS and NO and the inactivation of antioxidant enzymes (del Río, 2015). As a result, the concentration of these reactive species is suddenly elevated, which is necessary to trigger specific cellular responses. These responses include defense mechanisms to abiotic stresses, such as increased concentration and activity of antioxidant systems (Shi et al., 2014) or programmed cell death, which is important to eliminate cells that have been severely damaged (Yun et al., 2011). The enzymes involved in the synthesis of ROS and NO can be found in different cellular compartments (**Figure 1**), and their temporal and spatial localization is critical for signaling (Groß et al., 2013). Indeed, ROS and NO have unique roles based on their compartment of origin, which is probably due to interactions with local molecules in each organelle (Møller and Sweetlove, 2010; Shapiguzov et al., 2012; Mur et al., 2013). It has been observed, for example, that the transcriptional changes mediated by H_2_O_2_ produced in the apoplasts are distinct from the gene expression responses triggered by H_2_O_2_ produced in the chloroplasts (Gadjev et al., 2006; Sierla et al., 2013). Similarly, the NO generated from the plasma membrane is important in hypoxic conditions, whereas the NO generated from the chloroplasts and mitochondria is involved in the response to heavy metals (Kumar and Trivedi, 2016).

The plasma membrane is the main site of ROS production due to activity from proteins belonging to the NADPH oxidase family (respiratory burst oxidase homolog, RBOH). NADPH oxidases are integral membrane proteins that promote the transfer of cytoplasmic NADPH electrons to extracellular oxygen, forming $O_{2}^{•-}$ and promoting ROS accumulation in the apoplast (Das and Roychoudhury, 2014). Several studies have shown that stressful conditions stimulate the expression and activity of NADPH oxidases, leading to an oxidative burst (Jajic et al., 2015; Wang X. et al., 2015). Other oxidases and peroxidases associated with the cell wall are also involved in the generation of ROS in the apoplast, although their involvement in the response to stressors is not well defined (Das and Roychoudhury, 2014). In addition to promoting specific signaling events, which involve interactions with local signals, the RBOH-mediated oxidative burst of ROS production triggers the production of ROS in neighboring cells, initiating a long distance signaling event called a ROS wave. Each cell along the ROS wave activates their own RBOH proteins, generating a systemic wave of propagation of ROS production, which travels through the apoplast from the initial tissue to whole plants at rates of up to 8.4 cm min^-1^, promoting systemic acquired acclimation (SAA; Mittler and Blumwald, 2015). SAA enables all plant cells, not just those who first perceived the external stimulus, to alter their gene expression and metabolism in response to the stressor. Although the ROS wave is necessary for SAA, the response elicited is not always specific to the stress that initiated the signaling process, suggesting that the main function of the ROS wave is to prepare the plant for SAA and that other signals are required to mediate stress-specific SAA (Gilroy et al., 2014).

In addition to the apoplast, various cellular organelles, such as chloroplasts and mitochondria, also generate ROS. In fact, when illuminated, chloroplasts are important sources of ROS due to the intense electron transport during photosynthesis and the release of oxygen in PSII (Gupta and Igamberdiev, 2015). In mitochondria, ROS production occurs when the transfer of electrons exceeds the capacity of the alternative oxidase and the cytochrome oxidase to eliminate excess electrons, resulting in their transfer to molecular oxygen, mainly from complexes I and III. Another organelle involved in ROS synthesis in stressful conditions is the peroxisome. Peroxisomes generate $O_{2}^{•-}$ and H_2_O_2_ as a result of their metabolic activity, which involves processes such as photorespiration, the glyoxylate cycle, and β-oxidation (Tripathy and Oelmüller, 2012). These different pools of ROS, produced in distinct compartments, communicate with each other in the cells to regulate the plant metabolism. It is believed, for example, that the signal generated by the oxidative burst in the apoplast is transduced to chloroplasts, where a second wave of ROS generation is initiated (Shapiguzov et al., 2012). This signal transduction probably involves cytosolic components as well as the transport of ROS through the lipid bilayer (the O_2_^-^ produced in the apoplast can be converted to H_2_O_2_, which enters the cell through the aquaporins) or signal detection by apoplastic proteins and membrane receptors (de Dios Barajas-López et al., 2013). ROS generated in the chloroplasts, in turn, are involved in the retrograde signal from the chloroplast to the nucleus and influence the expression of many defense genes, in addition to inhibiting the transcription of genes associated with photosynthesis (de Dios Barajas-López et al., 2013). This has also been observed in other organelles, such as peroxisomes, where ROS accumulation can alter gene transcription (Sandalio and Romero-Puertas, 2015).

The maintenance of ROS levels also involves the participation of antioxidant mechanisms, which are associated with the elimination of these reactive species and can be divided into enzymatic and non-enzymatic mechanisms. Among enzymatic antioxidants, superoxide dismutase (SOD) is especially important because it catalyzes the removal of $O_{2}^{•-}$, the first ROS formed after exposure to various stressors. Other antioxidant enzymes include ascorbate peroxidase (APX), glutathione peroxidase (GPX), and catalase (CAT), which convert H_2_O_2_ to water (Lázaro et al., 2013). In combination with these enzymes, non-enzymatic antioxidants, such as glutathione, ascorbate, and tocopherol, also play a crucial role in maintaining ROS levels by acting as redox buffers in plant cells. Although the synthesis of these antioxidant molecules may, at first, be inhibited to allow the occurrence of the oxidative burst (del Río, 2015), once the signal is initiated, these mechanisms are activated and function cooperatively (Viehweger, 2014).

In contrast to ROS, the mechanisms of NO synthesis in plant cells are not yet fully understood, constituting one of the major challenges to studies investigating this signaling molecule. However, several biosynthetic pathways for NO have been proposed (**Figure 1**), which can be divided into reductive pathways, including the action of xanthine oxidoreductase in the peroxisomes (Millar et al., 1998) and nitrite:NO reductase attached to the membrane (Stöhr et al., 2001); and oxidative pathways, such as the pathways mediated by hydroxylamines (Rümer et al., 2009) and polyamines (Filippou et al., 2013). Apparently, the action of nitrate reductase (NR), a cytosolic enzyme essential for the assimilation of nitrogen, also represents an important source of NO for plants (Horchani et al., 2011). It has been suggested that NR is involved in the production of NO during a variety of physiological processes, such as bacterial defense (Modolo et al., 2005; Mur et al., 2013), hypoxia (Igamberdiev and Hill, 2004), cold (Zhao et al., 2009), drought (Freschi et al., 2010), and various aspects of development, such as floral transition and the formation of lateral roots (Seligman et al., 2008; Mur et al., 2013). However, under normal growth conditions, NR preferentially reduces nitrate to nitrite, and NR is only able to generate significant amounts of NO under certain conditions, such as anaerobic conditions or high concentrations of nitrite (Gupta et al., 2011; Mur et al., 2013). There have been numerous reports of an arginine-dependent nitric oxide synthase (NOS) in extracts of different plant species (Jasid et al., 2006; Zhao et al., 2007; Gas et al., 2009; del Río, 2011), but its presence in plants has not been unequivocally demonstrated (Domingos et al., 2015; Gupta and Igamberdiev, 2015).

In addition to biosynthetic processes, another crucial factor in NO concentration in the cell is the formation of *S*-nitrosothiols, particularly *S*-nitrosoglutathione (GSNO), relatively stable molecules in solution that may act as reservoirs of NO (Leterrier et al., 2011). GSNO is formed by *S*-nitrosylation of glutathione (GSH) by NO and can be transported in the phloem, thus contributing to the transport of this signaling molecule over long distances, which plays an important role in SAA (Arasimowicz-Jelonek et al., 2014). GSNO also regulates the NO concentration in the cell via inhibition of the nitrogen assimilation pathways (Fungillo et al., 2014). GSNO turnover is controlled by GSNO reductase (GSNOR), which catalyzes the deamination of GSNO into glutathione disulfide (GSSG) and NH_3_. Thus, GSNOR regulates the cellular levels of GSNO and is important in maintaining homeostasis of NO, which is essential for transient cell signaling (Malik et al., 2011). The levels and activity of GSNOR are modulated in conditions of stress and are determined, among other factors, by the balance between ROS and NO (Cheng et al., 2015; Wang D. et al., 2015; Yang et al., 2015).

Degradation of NO is as important as synthesis and transport in determining the final concentration of this signal molecule in plant cells. Recently, Sanz-Luque et al. (2015) demonstrated that the green alga *Chlamydomonas reinhardtii* has a specific mechanism for the elimination of NO, which involves truncated hemoglobin THB1. The authors verified that THB1 has NO dioxygenase activity (produces NO_3_^-^ from NO and O_2_) and maintains in its active form through a mechanism that removes electrons from NR and alters its activity. Another class of hemoglobins, the non-symbiotic hemoglobins (nsHb), particularly those belonging to the GLB1 class, have also been reported to have NO dioxygenase activity and to promote the degradation of NO in certain circumstances, such as hypoxia (Perazzolli et al., 2004). The reduction in *GLB1* expression, moreover, allows NO concentration to increase, triggering defense responses against stress (Mur et al., 2012). The interaction between NO and $O_{2}^{•-}$, which generates peroxynitrite (ONOO^-^), is also regarded as a mechanism of NO elimination and involves the modulation of mitochondrial activity (Wullf et al., 2009).

The biosynthesis and degradation of ROS and NO influence each other. ROS are well-known inducers of NO synthesis in various plant species exposed to abiotic stress, although the signaling involved in this process is still not completely understood. NO, in turn, limits the accumulation of ROS by inhibition of NADPH oxidases, as well as by promoting changes in the antioxidant systems, suggesting the existence of complex feedback regulation of both signaling molecules (Groß et al., 2013). In fact, the activation of antioxidant mechanisms to maintain ROS homeostasis often involves the participation of NO (Farnese et al., 2013; Shi et al., 2014; Silveira et al., 2015). It has been shown that the addition of NO increased the activity of SOD up to 110% in sorghum plants exposed to arsenic (Saxena and Shekhawat, 2013), in addition to the increase in CAT and APX and the activation of the ascorbate-glutathione cycle (Hasanuzzaman and Fujita, 2013; Shi et al., 2014; Cheng et al., 2015). Some studies, however, have suggested that NO can inhibit the antioxidant capacity of the cell (Marti et al., 2013). These seemingly contradictory results may be due to the dose-dependent effects of NO on cellular redox status. According to this hypothesis, low concentrations of NO stimulate the antioxidant system and promote adaptation to stress conditions, while high concentrations of NO trigger severe cell damage and even cell death (Thomas et al., 2008; Groß et al., 2013).

## Molecular Bases of ROS and NO Action

The mode of action of ROS and NO at the molecular level was and still is the subject of many studies, both in plants and other organisms, such as mammals and bacteria (Green et al., 2014; Lamotte et al., 2015; Morales et al., 2015). The available data indicate that the effects of NO, as well as certain species derived from this molecule, depend on chemical changes in proteins, which can occur by three different mechanisms: metal nitrosylation, tyrosine nitration, and *S*-nitrosylation (Lamotte et al., 2015). Metal nitrosylation consists of NO binding to transition metals in metalloproteins. Soluble guanylate cyclase is an example of an enzyme that is modulated by this type of post-translational modification. Tyrosine nitration is the addition of a nitro group to tyrosine residues. Tyrosine nitration is carried out mainly by peroxynitrite (ONOO^-^), the product of the reaction between NO and $O_{2}^{•-}$. Although tyrosine nitration was originally considered indicative of stress, recent evidence suggests its involvement in cell signaling (Mengel et al., 2013). Finally, *S*-nitrosylation, which consists of NO binding to cysteine residues in target proteins, is apparently the principal mechanism for the transduction of the NO bioactivity. *S*-nitrosylation can also occur via *trans*-nitrosylation, that is, by the transfer of NO from an *S*-nitrosylated residue to another thiol group through the action of low-molecular weight nitrosothiols, such as GSNO (Lamotte et al., 2015). Regardless of the mechanism involved, *S*-nitrosylation is a post-translational modification that can alter the activity, stability, conformation, interactions with other molecules or subcellular localization of the target protein, regulating a wide range of cellular functions and signaling events (Sevilla et al., 2015).

*S*-nitrosylation is an important process in plant responses to abiotic stress. Exposure to salt stress, for example, results in the *S*-nitrosylation of enzymes involved in different physiological processes, such as respiration, photorespiration, and antioxidant pathways (Camejo et al., 2013), while in plants exposed to low temperatures, the enzymes involved in carbon metabolism were the main group of *S*-nitrosylated proteins (Puyaubert et al., 2014). *S*-nitrosylation of proteins that participate in central processes in the plant cell presumably contributes to the metabolic reprogramming required to maintain homeostasis under stress conditions. In addition to changes in cellular enzyme dynamics, *S*-nitrosylation may also trigger changes in gene expression as a result of *S*-nitrosylation of transcription factors, affecting their affinity for DNA or their location. Recently, it was demonstrated that *S*-nitrosylation is a negative regulator of transcription factors from the MYB family (regulator of tolerance to biotic and abiotic stresses), which may be important for the inactivation of this regulatory protein after the initial response of plants to stress (Tavares et al., 2014). Several *S*-nitrosylated nuclear proteins have also been identified, including histone deacetylases, which highlights the regulatory role of NO in events located in the nucleus (Chaki et al., 2015). Histone deacetylases are responsible for the removal of acetyl groups on histones, promoting the chromatin condensation, which makes the genes less accessible to the transcriptional machinery (Mengel et al., 2013). In mammalian cells, *S*-nitrosylated histone deacetylases become detached from the chromatin, increasing acetylation and gene expression (Nott et al., 2008). Thus, the *S*-nitrosylation of deacetylases suggests that NO participates in the regulation of epigenetic processes in plants (Floryszak-Wieczorek et al., 2012; Chaki et al., 2015).

The *S*-nitrosylation state of any protein is determined by the balance between nitrosylation and denitrosylation reactions. In fact, denitrosylation, which involves the removal of NO from cysteine residues, is essential for the reversibility of *S*-nitrosylation and influences the enzyme activity, protein–protein interactions and many other aspects of signaling (Sevilla et al., 2015). Although this process has been more extensively studied in mammals, recent evidence has shown that it occurs in plant cells (Kneeshaw et al., 2014; Benhar, 2015). Among the molecules which may be involved in the denitrosylation process, the GSNO/GSNOR (discussed earlier in this review) and the thioredoxin/thioredoxin reductase (Trx/TR) systems are essential for the maintenance of homeostasis of nitrosothiols in plants (Lamotte et al., 2015). In the Trx/TR system, Trx reduces nitrosothiols through its dithiol moiety, generating free thiol groups and oxidized Trx. Regeneration of Trx occurs through the action of TR and NADPH (Benhar, 2015). Thus, the control of the redox status of thiol groups depends on their interaction with NO and with the denitrosylation systems, which influences the intensity and duration of the signaling events (Benhar, 2015).

As observed for NO, ROS also transmit signals via post-translational modifications in proteins and, once more, cysteine residues are the main targets. However, while NO promotes *S*-nitrosylation, ROS can trigger a diverse range of oxidative post-translational modifications (Ox-PTM), reversible or irreversible, including *S*-glutathionylation, disulfide bond formation, and sulfhydration (Akter et al., 2015). A single protein can undergo different types of Ox-PTMs, and there is evidence that each Ox-PTM may have a distinct biological role (Couturier et al., 2013). The first step in ROS-dependent signaling involves the reverse oxidation of a cysteine residue, forming sulfenic acid (R-SOH). This modification is highly unstable and will lead to subsequent changes; the major ones are the reaction with free protein thiols to form disulfide bonds or the covalent attachment of low-molecular weight thiols, such as GSH, promoting *S*-glutathionylation, a process that is important in signaling and protein protection against superoxide. The reduction of disulfide bonds and deglutathionylation interrupt the signal that initiated with the Ox-PTMs and are controlled by glutaredoxins and thioredoxin, respectively (Waszczak et al., 2014, 2015).

The Ox-PTMs, particularly *S*-glutathionylation, play a central role in the response to abiotic stresses and can modulate numerous cellular processes affecting proteins, transcription factors, and chromatin structure. These mechanisms, however, have mostly been studied in animals and bacteria, and many aspects of the Ox-PTM-mediated responses are unknown in plants (Zagorchev et al., 2013). One example of Ox-PTMs mediating changes in cellular dynamics is the transcription factor ERFVII (ethylene-responsive group factor VII), which is important in altering gene expression under hypoxic conditions. ERFVII is bound to the plasma membrane and is only released in low-oxygen conditions. Its translocation to the nucleus activates the expression of hypoxia-responsive genes. In the presence of oxygen, however, ERFVII cysteine residues are oxidized to sulfenic acid, conjugated with arginine and directed to degradation, down-regulating the expression of genes that are no longer needed (Dietz, 2014). The Ox-PTMs can also be positive regulators of gene transcription, as in the case of transcription factors of heat shock proteins (HSF), whose oxidation by H_2_O_2_ induces translocation from the cytosol to the nucleus (Habibi, 2014). Despite the growing number of studies, however, there is still little information about the effects of *S*-nitrosylation and Ox-PTMs on gene expression and the consequences of these changes on plant metabolism in stress conditions. Thus, the molecular mechanisms involved in cell signaling mediated by ROS and NO are still far from being fully understood.

## Crosstalk Between ROS and NO in the Response to Abiotic Stress

### Heavy Metals

Traditionally, heavy metals are considered those chemicals that have a density higher than 5 g cm^-3^ or an atomic number higher than 20. In plant physiology, however, the term heavy metal has been used generically to refer to any metal or metalloid that is toxic to plants, even when present at low concentrations (Singh et al., 2011; Oz et al., 2015). Although some of the members of this group are necessary for growth and development, others have no known function in plant cells and, regardless of their physiological role, the accumulation of metals usually results in severe cell damage, which can lead to the death of the plant (Besson-Bard et al., 2009). Heavy metals enter plant cells by transporters present in the plasma membrane and may be retained in the roots or transported to the shoots, according to the cellular detoxification mechanisms in each species (Tangahu et al., 2011). In general, the defense mechanisms of plants to heavy metals can be divided into two groups, which may occur simultaneously: (i) regulation of the concentration of free metal in the cytosol through metal exclusion, metal binding to the cell wall or compartmentalization in the vacuole; and (ii) physiological, biochemical, and molecular changes that allow the reprogramming of plant metabolism and the maintenance of cellular homeostasis.

**FIGURE 2:**
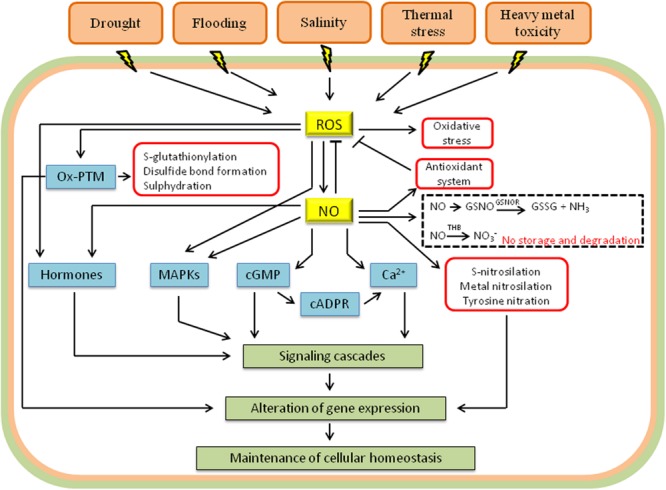
**Schematic representation showing the interplay between NO, ROS, and other signaling molecules in response to abiotic stress.** The signaling molecules include hormones, mitogen-activated protein kinases (MAPKs), cyclic guanosine monophosphate (cGMP), cyclic adenosine diphosphoribose (cADPR) and calcium (Ca^2+^). Besides the interaction with components of signaling pathways, NO and ROS also transmit signals via post-translational modifications in proteins [metal nitrosylation, tyrosine nitration, *S*-nitrosylation, and oxidative post-translational modifications (Ox-PTM)]. ROS degradation by antioxidants and NO storage (GSNO, *S*-nitrosoglutathione) and degradation by GSNOR (GSNO reductase) and THB (truncated hemoglobin) are demonstrated. *Arrows* and *T-bars* indicate activation and inhibition, respectively (adapted from Oz et al., 2015).

Recent studies have indicated that the interaction between NO and ROS is essential for tolerance to heavy metals (**Figure 2**) (Wang et al., 2014; Feigl et al., 2015; Silveira et al., 2015; Thao et al., 2015). The exact signaling mechanisms involved in this process, however, have not been clarified, and many questions remain unanswered. Currently, it is known that increases in ROS are one of the first cellular signals in response to excess heavy metals. In fact, heavy metals can activate the production of ROS in the apoplast, stimulating NADPH oxidases, and in organelles such as chloroplasts and mitochondria (Chmielowska-Bak et al., 2014). Heavy metals enhance the synthesis of NO by mechanisms that vary according to the chemical characteristics of the metal. Lead, for example, apparently increases the activity of cytosolic NR (Yu et al., 2012), while increases in NO levels mediated by cadmium are related to iron-induced deficiency (Besson-Bard et al., 2009). Interestingly, some metalloids, such as arsenic, stimulate both the synthesis of NO and the activity of GSNOR, and the balance between these two processes will determine the final concentration of the signaling molecule (Leterrier et al., 2012). In addition, the increase in NO may result from signaling triggered by excessive ROS following exposure to metals (Yun et al., 2011).

According to their concentrations, ROS and NO can cause oxidative/nitrosative stress in cells or may act as signaling molecules. At low concentrations, NO contributes to increased tolerance of the plants to metals in various ways, for example, by promoting metal binding to the cell wall, preventing their entry into the cell (Singh et al., 2011) or promoting their compartmentalization in the vacuole, either by increasing phytochelatin synthesis (De Michele et al., 2009) or by altering the activity of proton pumps in the vacuolar membrane to create an electrochemical gradient that favors the absorption of metals (Cui et al., 2010). In addition to these effects, NO can also reprogram plant physiological processes and stimulate the synthesis and activity of antioxidant systems, which is essential to limit the oxidative stress induced by metals (Cheng et al., 2015; Andrade et al., 2016). Finally, post-translational changes triggered by NO can decrease the activity of enzymes involved in ROS metabolism, such as glycolate oxidase and NADPH oxidase, allowing the cell to re-establish redox homeostasis (Yun et al., 2011; Quiang et al., 2012).

### Drought

Water deficits are the main environmental factor limiting the growth and productivity of plants worldwide. Indeed, the damage triggered by drought may be greater than the damage caused by other biotic and abiotic factors combined (Chaves et al., 2009). Plants tolerant to drought usually have a strict control of stomatal movements and a fine balance of cellular metabolism, and both ROS and NO are important in these processes (**Figure 2**) (Osakabe et al., 2014). Drought stress-induced NO is found in a wide variety of plant species, including vegetables, horticultural plants and epiphytes, suggesting the universal requirement of NO during drought stress signaling (Santisree et al., 2015). The metabolic pathways involved in this process, however, are still unclear, although evidence suggests the involvement of NR (Arasimowicz-Jelonek et al., 2009) and xanthine oxidoreductase (Yu et al., 2014). The generation of ROS during drought, on the other hand, is well known and commonly involves changes in plant metabolic processes. For example, drought may reduce the activity of Rubisco (Parry et al., 2002), which compromises the fixation of CO_2_ and the regeneration of NADP^+^ via the Calvin cycle. This results in an over-reduction of the electron transport chain in the chloroplasts and, consequently, the leakage of electrons to O_2_, mainly by the Mehler reaction and photorespiration, resulting in the generation of ROS (Carvalho, 2008). In parallel with the physiological changes, NADPH oxidases are also involved in the generation of ROS in water stress conditions, as they are essential for the activation of defense responses against drought (Wang X. et al., 2015).

One of the first and most important physiological responses induced by drought is the reduction of the stomata opening (Neill et al., 2008). In water stress conditions, the complex dynamics of stomatal movement are directly related to the concentration of abscisic acid (ABA), ROS, and NO. In this process, ABA, an important plant hormone traditionally associated with responses to water stress, acts as an upstream regulator, inducing the synthesis of NO. NO and ROS, in turn, act synergistically to mediate stomatal closure through the formation of 8-nitro-cGMP (Joudoi et al., 2013). ABA is also involved in several other plant responses to water stress, for example, the induction of gene expression and the synthesis of defense compounds, such as proline. Increasing only ABA, however, is not sufficient to induce the synthesis of these compounds, as observed after exogenous application of ABA to *Arabidopsis thaliana* (Verslues and Bray, 2006), suggesting that factors other than ABA are required to modulate the response to drought, possibly the cellular redox status. Thus, the production of ROS and the subsequent change in the redox state of the cell have been suggested as factors required to initiate signal transduction mediated by ABA (Carvalho, 2008).

Similar to ABA and ROS, NO has been shown to alter gene expression in response to water deficits. Transgenic *Arabidopsis* lines that constitutively express rat neuronal NO synthase showed changes in gene expression relative to wild plants when subjected to drought, with 184 genes up-regulated and 263 down-regulated. The main transcriptional changes were observed in the genes involved in redox metabolism and sugar metabolism and in transcription factors. These transcriptional changes were accompanied by higher survival rates and high biomass production, indicating a protective effect of NO (Shi et al., 2014). Moreover, it has been reported that NO may trigger epigenetic modifications, such as DNA methylation, in response to water stress conditions. Indeed, it was observed that the exogenous application of SNP (an NO donor) decreases the overall methylation levels in *Dendrobium huoshanense*, leading to increases in the activity of antioxidant enzymes (Fan et al., 2012).

### Flooding

Climate projections for the next decades, besides pointing out an increase in drought intensity and frequency for some regions, also highlight a significant increase in rainfall for others, especially those regions with tropical climate (Hirabayashi et al., 2013). If these predictions are confirmed, productivity for several crop areas and ecosystems can be significantly impacted due to flooding events (Bailey-Serres and Voesenek, 2008). Despite the fact that flooding has been studied to a lesser extent than drought, in recent years, researches about molecular mechanisms that promote flooding tolerance have progressed rapidly (Voesenek and Bailey-Serres, 2015). In general, the mechanisms that confer tolerance to such stress can be grouped into two categories: “escape,” which involves anatomical and morphological modifications that allow access of the submerged cells to O_2_ and CO_2_, and “quiescence,” which promotes a profound change in metabolism and growth of flooded plants (Voesenek and Bailey-Serres, 2015).

Several evidences have suggested that plants can sense the reduction of O_2_ availability caused by flooding through different ways. Some of these mechanisms of perception may involve changes in the dynamics of ROS and NO production and consumption, because of mitochondrial electron transport inhibition (Rhoads and Subbaiah, 2007). In fact, studies in *Arabidopsis thaliana* showed that oxygen deprivation induces ROS production in the complex III, which triggers a transient activation of MAPK signaling cascade (Chang et al., 2012). In addition, regulatory responses induced by ROS involve, at least in part, the interaction with ethylene, an important plant hormone involved in plant response to various stresses. Indeed, in rice plants, ROS and ethylene were involved in the production of adventitious root in stem nodes (Steffens et al., 2012).

The origins of NO in hypoxic conditions are not clearly understood, although nitrite and ascorbate are apparently involved in this process, as well as mitochondrial reactions (Wang and Hargrove, 2013). NO generation in mitochondria is an important process at low oxygen conditions. In this process, the nitrite acts as an electron acceptor in complex IV, complex III and in the alternative oxidase, keeping a limited production of ATP when oxygen is not available and, thus, preventing the collapse of cellular energy status (Gupta et al., 2011). The produced NO diffuses into the cytosol, where it is converted to nitrate by hemoglobin action. Class 1 hemoglobins are proteins capable of binding to NO, and at low O_2_ concentrations catalyze its conversion to nitrate, which can be converted to nitrite by NR (Dordas, 2015). Nitrite, in turn, can be converted again to NO in the mitochondria, closing the cycle (Gupta et al., 2011).

### Salt Stress

Salinity is an ever-present threat to crop yields, especially in places where irrigation is required. In fact, studies show that approximately 20% of all cultivated areas in the world are affected by this type of stress, and this percentage is likely to increase due to inadequate irrigation practices (Muns and Tester, 2008). Salt stress compromises the intracellular ion homeostasis, which leads to membrane dysfunction, alteration of metabolic activities, growth inhibition, and even cell death (Zhang et al., 2006). Plants tolerant to salt stress have many diverse strategies to tolerate high concentrations of solutes. The principal mechanisms include, but are not limited to, (i) ion homeostasis and compartmentalization, (ii) ion transport and uptake, (iii) biosynthesis of osmoprotectants and compatible solutes, (iv) activation of antioxidant enzymes and synthesis of antioxidant compounds, and (v) synthesis of polyamines (Gupta and Huang, 2014). All these events are triggered and integrated into the plant cell through the action of signaling molecules, especially ROS, NO, and plant hormones (**Figure 2**) (Filippou et al., 2014).

Nitric oxide is essential for the tolerance of plants to high salt concentrations. Examples of the role of NO in this process can be observed in mutant plants of *Arabidopsis* (*Atnoa1*) which show deficiency in NO synthesis and hypersensitivity to salt stress (Zhao et al., 2007). Additionally, studies using NO donors and inhibitors showed that the ability of NO to alleviate stress was related to the change in the Na^+^/K^+^ ratio in the cytosol due to the increase in H^+^-ATPase and H^+^-PPase activity in the plasma and vacuolar membranes (Zhao et al., 2004; Zhang et al., 2006; Wang et al., 2009). In addition, NO is also able to induce the expression of defense genes against stress and significantly increase the activity of enzymes of the antioxidant system (Uchida et al., 2002). H_2_O_2_ also alters the expression of stress response genes in plants subjected to high salt concentrations, particularly in root cells, in addition to increasing the activity of specific enzymes (Miller et al., 2010). It is also important to note that the changes triggered by ROS and NO are not restricted to the tissues where these molecules were produced because addition of NO and H_2_O_2_ to the roots reduced the physiological imbalances caused by NaCl in leaves of *Citrus* plants (Tanou et al., 2009). This process is possible due to mechanisms such as the ROS wave and the transport of NO because both NO and GSNO have been observed in vascular tissues of plants exposed to salinity stress (Valderrama et al., 2007).

Another effect of NO and H_2_O_2_ in plants is the acquisition of immunity against salinity. This process, known as priming, describes the phenomenon in which plants previously subjected to a particular stress factor accelerate and potentiate their defense responses when subjected to that same stressor (Zhu, 2003; Zhang et al., 2004; Molassiotis et al., 2010). In fact, plants treated with low concentrations of NO and H_2_O_2_ had their metabolic and physiological responses potentiated when exposed to salt stress. Likewise, NO-associated salt priming could be observed in halophytes and glycophytes that have increased tolerance to salinity when previously exposed to NO donors (Li et al., 2005). These data show that NO and H_2_O_2_ are priming agents that promote increased tolerance in the whole plant, minimizing the deleterious effects of subsequent exposure to salinity.

As noted in this review, different abiotic stresses have the same convergence point: they induce the production of ROS and NO. Thus, although each type of stress has its own specific characteristics, nearly all abiotic stressors alter the cellular redox state, and therefore, the participation of antioxidants in the plant response is essential. The activity and gene expression of antioxidants are strongly influenced, directly or indirectly, by changes in the concentrations of ROS and NO (Shao et al., 2008; Shi et al., 2014). In addition to the well-established role of the antioxidants in removing ROS, which has been discussed here, these molecules are important in signaling, providing essential information about the cellular redox status and influencing the expression of defense genes against biotic and abiotic stresses (Pastori et al., 2003; Foyer, 2005). Moreover, antioxidants are also needed to interrupt the signal transduction cascades when they are no longer needed, as is the case of glutaredoxin and thioredoxin (Waszczak et al., 2015).

### Temperature Stress

Temperature, in combination with water deficiency, is one of the main abiotic factors that determine the survival and distribution of species worldwide. There are several reports in the literature indicating the adverse effects of high and low temperatures on the molecular, biochemical, and physiological characteristics of plants (Suzuki and Mittler, 2006). Although exposure to high and low temperatures triggers very distinct metabolic disorders, there is a common response between these two stressors: the increase in ROS, which damages cellular structures (**Figure 2**) (Potters et al., 2007; Zhou et al., 2012; Bita and Gerats, 2013). Similar to other abiotic stresses, increased levels of ROS following extreme temperatures appear to involve an imbalance between capturing and processing energy (Hasanuzzaman et al., 2013). However, over the past few years, several studies have shown that ROS and RNS, especially the interaction between them, are essential for acclimatization to high and low temperatures (Yu et al., 2014; Hossain et al., 2015). In fact, plants treated with low doses of NO and H_2_O_2_ perform better under conditions of thermal stress (Neill et al., 2002; Uchida et al., 2002; Abat and Deswal, 2009; Cantrel et al., 2011).

Exposure to high temperatures usually results in increased production of NO, a response that is important for acclimation to this type of stress (Leshem, 2000; Neill et al., 2003; Bouchard and Yamasaki, 2008; Yu et al., 2014). Indeed, addition of exogenous NO to plants subjected to high temperatures promotes the activation of enzymatic and non-enzymatic defense systems against ROS, reducing cellular damage (Song et al., 2006; Zhao et al., 2009; Hasanuzzaman et al., 2013). In addition, treatment with NO scavengers, such as cPTIO, reverses the beneficial effects caused by NO, which further reinforces the importance of this molecule in the tolerance to high temperatures (Song et al., 2006). Similarly, accumulation of ROS, especially H_2_O_2_, has been shown to be involved in signal transduction that culminates in increased expression of heat shock genes, which encode proteins that play critical roles in the maintenance of cellular homeostasis in stressful conditions (Königshofer et al., 2008).

As observed in high temperatures, exposure to cold can also promote a rapid increase in the endogenous levels of NO and ROS (Zhao et al., 2009; Cantrel et al., 2011). The increased production of NO in these conditions most likely involves an increase in NR because the *nia1nia2* mutants (NR-defective double mutants) showed lower concentrations of NO and increased susceptibility to cold stress (Cantrel et al., 2011). Tolerance to low temperatures mediated by NO involves the reprogramming of gene expression because it has been shown that NO production, induced by NR, promotes the transcription of the *P5CS1* and *ProDH* genes, along with accumulation of proline and an increased tolerance to cold (Zhao et al., 2009). In addition to the effects mediated by NO, the role of H_2_O_2_ in cold acclimation has also been widely documented (Prasad et al., 1994; Yu et al., 2003; Hung et al., 2007; Wang et al., 2010). Several studies indicate that exogenous application of H_2_O_2_ reduces cellular damage caused by low temperatures, increasing the survival rates (Neill et al., 2003). Again, this beneficial effect of H_2_O_2_ appears to involve an increase in the effectiveness of both the enzymatic (increased activity of the enzymes APX, GPX, and CAT) and non-enzymatic antioxidant mechanisms (increases in GSH levels; İseri et al., 2013; Wang et al., 2013). The signaling cascade responsible for the attenuation of cold stress involves, at least partially, transient increases in the cytosolic Ca^2+^ concentration due to H_2_O_2_-mediated activation of Ca^2+^ channels in the plasma membrane (Knight et al., 1996). However, although these results clearly indicate that ROS and NO are key molecules in the tolerance to high and low temperatures, few studies have focused on the molecular aspects and the signaling cascades responsible for these processes.

## Interactions Between ROS, NO, and Other Signaling Molecules in the Response to Abiotic Stress

The signaling pathways that respond to environmental stresses constitute intricate molecular networks that involve many other components besides ROS and NO, such as calcium (Ca^2+^), cyclic nucleotides, plant hormones, and mitogen-activated protein kinases (MAPKs; **Figure 2**). The MAPK cascades, for example, are activated by ROS, NO, and hormones, representing a convergence point for these signaling molecules. MAPK signal transduction involves a phosphorylation cascade comprising MAPK kinase kinase (MAPKKK), MAPK kinase (MAPKK), and finally, MAPK, which is activated after phosphorylations of threonine and tyrosine residues in the conserved motif T-X-Y (Rodriguez et al., 2010). The interaction between ROS, NO, and MAPKs has been demonstrated in the plant response to various stressors, such as heavy metals (Chmielowska-Bak et al., 2014), drought (Wang et al., 2014), and osmotic stress (Xu et al., 2011). In *Arabidopsis*, high levels of ROS, especially H_2_O_2_, induce the transcription of the genes *OXI1* (Rentel et al., 2004) and *ANP1* (Kotvun et al., 2000), which encode protein kinases required for full activation of the MAPKs. NO acts simultaneously with ROS in the activation of MAPKs, although the mechanisms involved in this process are not completely understood. In mammals, the activation of MAPK cascades by NO occurs indirectly and involves an increase in the synthesis of cGMP (Francis et al., 2010). It is possible that a similar mechanism is present in plants because it has been demonstrated that NO triggers the increases in the concentration of cyclic nucleotides (Pasqualini et al., 2009). Once active, MAPKs can phosphorylate many target molecules, both in the cytosol and in the nucleus, including enzymes or transcription factors (Rodriguez et al., 2010). The responses mediated by MAPKs involve induction of antioxidative or pro-oxidative enzymes and may attenuate or amplify the original signal triggered by ROS and NO (Asai et al., 2008; Opdenakker et al., 2012). In addition, MAPKs may also interfere in the signaling and in the biosynthesis of hormones, leading to the activation of downstream stress responses. The signal is interrupted by phosphatases, which promote the dephosphorylation of MAPK (Opdenakker et al., 2012).

Plant hormones are also critical messengers in plant development and stress tolerance. The signaling cascades of most hormones include interactions with ROS and NO, as is the case of ABA, mentioned above, and auxin. Auxin is an essential plant hormone that participates in various cellular processes. In certain situations, auxin induces the synthesis of ROS and NO, which, in turn, influence the hormone-mediated signaling (Joo et al., 2001; Yadav et al., 2013). ROS accumulation in stressful situations can trigger oxidative inactivation or degradation of auxin, as well as a decrease in the expression of genes involved in its transport and signaling, through specific MAPK cascades (Xia et al., 2015). GSNO accumulation also compromises the polar transport of auxin and reduces its effects via *S*-nitrosylation of components of its signaling pathway (Shi et al., 2015). The attenuation of auxin signaling leads to changes in plant growth and acclimatization to new environmental conditions. In some cases, however, ROS and NO can act as positive regulators of auxin (Woodward and Bartel, 2005; Terrile et al., 2012). Another hormone involved in the acclimation of plants to stress is salicylic acid (SA), which is responsible for transcriptional reprogramming during the defense against abiotic stress. The interaction between SA, ROS, and NO is complex, with ROS and NO acting both upstream and downstream of SA. NO and ROS induce the synthesis of SA, and NO also activates transcription factors that initiate SA-dependent gene expression by inducing the synthesis of various molecules, such as pro-oxidants and antioxidants (Mur et al., 2013). The synthesis of pro-oxidants and antioxidants during SA-mediated signaling presents biphasic redox dynamics. In the first phase, the oxidative phase, transient increases in ROS levels trigger signaling events that are dependent on the cell redox state, and this is followed by a reductive phase characterized by increased antioxidants and decreased ROS (Herrera-Vásquez et al., 2015). In addition to ABA, auxin, and SA, several other hormones, such as ethylene, gibberellins, and brassinosteroids, interact with ROS and NO in the process of acclimation to stress (Bartoli et al., 2013; Xia et al., 2015). These hormones also interact with each other and with other cellular messengers, such as Ca^2+^, and the final response is dependent on the fine balance between all of these components (Xia et al., 2015).

In animals, NO-mediated signaling is largely dependent on the synthesis of cyclic nucleotides, especially cyclic GMP (cGMP). In these organisms, NO binding to a soluble guanylate cyclase increases the activity of this enzyme up to 200 times, with a consequent increase in the formation of cGMP (Francis et al., 2010). In plants, both NO and H_2_O_2_ increase the concentration of cyclic nucleotides, which apparently involves the binding of these molecules to enzymes with guanylate cyclase activity (Dubovskaya et al., 2011; Mulaudzi et al., 2011). In *Chlamydomonas*, an NO-dependent guanylate cyclase that participates in the transcriptional repression of NR has been identified, suggesting a model that integrates, among other components, NO, cGMP and the nitrogen assimilation pathways (de Montaigu et al., 2010). Two different types of responses are associated with cGMP, depending on the time between the perception of the stimulus and the peak in nucleotide concentration: fast responses, which involve the modulation of ion channels, such as Ca^2+^ channels; and long-term adaptive responses, which result in changes in the transcriptome and in the proteome (Pasqualini et al., 2009). In addition to increasing the concentration of cGMP (Durner et al., 1998; Dubovskaya et al., 2011), ROS and NO also promote nitration of this molecule, changing its structure and function, and the stomatal closure is one of the clearest examples of the interaction of NO, ROS, and cGMP (Joudoi et al., 2013).

Ca^2+^ is a highly versatile signaling molecule that plays a central role in the response to environmental stressors (Dodd et al., 2010; Schulz et al., 2013; Chmielowska-Bak et al., 2014). The exposure to stressors activates calcium channels, pumps and transporters in the plasma membrane or in the membranes of organelles, which results in the rapid influx of the cation into the cytosol, increasing the concentration of cytosolic Ca^2+^. Transient calcium levels in the cytosol are detected by calcium-binding proteins such as calmodulin, Ca^2+^-dependent protein kinases (CDPKs), phosphatases regulated by Ca^2+^ and by changes in Ca^2+^ channels and pumps (Gilroy et al., 2014), and these signals are then transmitted. One example is the Ca^2+^-permeable channels in the plasma membrane, which are relatively inactive under normal conditions but can be activated by ROS in the guard cells in response to drought (Pei et al., 2000; Dodd et al., 2010). Similar to ROS, NO synthesis is induced by Ca^2+^, and it activates intracellular Ca^2+^-permeable channels and CDPKs (Astier et al., 2010).

## Future Perspectives

It is widely recognized that ROS and NO interact with each other and are key molecules in the plant response to various types of abiotic stresses. Additionally, a large body of evidence has shown that cellular redox signaling contributes to the development of SAA in plants and, in some cases, priming, which can involve other signaling networks, such as hormones and MAPK cascades. However, although there has been significant progress in elucidating the interplay between ROS and NO, many challenges remain. There is still little information, for example, on the initiation of the signaling mediated by ROS and NO, the mechanisms involved in the perception and the specificity of the generated signal, and the control of the delicate balance between production and scavenging of the reactive species. Detailed studies investigating cross-talk regulation among ROS, NO, hormones, cyclic nucleotides, MAPKs, and other signaling molecules are needed to clarify how these molecules interact with each other during different types of stresses.

Data obtained in recent years have shown that both ROS and NO trigger post-translational modifications of proteins, an important process for signal transduction. In fact, recent research analyzed the impact of ROS and NO on the *S*-nitroso-proteome, or redox proteome, of plants exposed to various stressors. In addition to these studies, further biochemical and functional characterizations of proteins that have been post-translationally modified are needed to provide a more comprehensive understanding of the effects of ROS and NO at the molecular level. It is also necessary to evaluate in more detail how these signaling molecules alter gene expression by analyzing, for example, their possible involvement in epigenetic processes. Finally, it is important to note here that many studies examining ROS and NO signaling used exogenous sources of NO or substances that induce the generation of H_2_O_2_. Despite the clear relevance of these studies, it is important to analyze ROS and NO naturally produced in response to the environment, thus contributing to understanding the signaling process *in planta*.

## Author Contributions

FF outlined the manuscript together with JO and wrote the following topics: biosynthesis, metabolism, and transport; molecular bases of ROS and NO action; and perspectives. PM-S wrote all the part about the different kinds of abiotic stresses (drought, hypoxia, salt stress, thermal stress, and heavy metal). GG wrote the introduction and the topic “Interaction among ROS, NO, and Other Signaling Molecules.” JO outlined the manuscript together with FF, guided and led the discussions and made a critical review of all the manuscript.

## Conflict of Interest Statement

The authors declare that the research was conducted in the absence of any commercial or financial relationships that could be construed as a potential conflict of interest.

## References

[B1] Abat J. K. Deswal R. (2009). Differential modulation of S-nitrosoproteome of *Brassica juncea* by low temperature: change in S-nitrosylation of Rubisco in responsible for the inactivation of its carboxylase activity. *Proteomics* 9 4368–4380. 10.1002/pmic.200800985 19655309

[B2] Akter S. Huang J. Waszczak C. Jacques S. Gevaert K. Van Breusegem F. (2015). Cysteines under ROS attack in plants: a proteomics view. *J. Exp. Bot.* 66 2935–2944. 10.1093/jxb/erv044 25750420

[B3] Andrade H. M. Oliveira J. A. Farnese F. S. Ribeiro C. Silva A. A. Campos F. V. (2016). Arsenic toxicity: cell signalling and the attenuating effect of nitric oxide in *Eichhornia crassipes*. *Biol. Plant.* 60 173–180. 10.1007/s10535-015-0572-4

[B4] Arasimowicz-Jelonek M. Floryszak-Wieczorek J. Drzewiecka K. Chmielowska-Bak J. Abramowski D. Izbianska K. (2014). Aluminum induces cross-resistance of potato to *Phytophthora infestans*. *Planta* 239 679–694. 10.1007/s00425-013-2008-8 24346311 PMC3928512

[B5] Arasimowicz-Jelonek M. Floryszak-Wieczorek J. Kubis J. (2009). Involvement of nitric oxide in water stress-induced responses of cucumber roots. *Plant Sci.* 177 682–690. 10.1016/j.plantsci.2009.09.007

[B6] Asai S. Ohta K. Yoshioka H. (2008). MAPK signaling regulates nitric oxide and nadph oxidase-dependent oxidative bursts in *Nicotiana benthamiana*. *Plant Cell* 20 1390–1406. 10.1105/tpc.107.055855 18515503 PMC2438462

[B7] Astier J. Besson-Bard A. Wawer I. Parent C. Sumaira R. Sylvain J. (2010). Nitric oxide signalling in plants: cross-talk with Ca2+, protein kinases and reactive oxygen species. *Annu. Plant Rev.* 42 147–170. 10.1002/9781444328608.ch7

[B8] Bailey-Serres J. Voesenek L. A. C. J. (2008). Flooding stress: acclimations and genetic diversity. *Annu. Rev. Plant Biol.* 59 313–339. 10.1146/annurev.arplant.59.032607.092752 18444902

[B9] Bartoli C. G. Casalongué C. A. Simontacchi M. Marquez-Garcia B. Foyer C. H. (2013). Interactions between hormone and redox signaling pathways in the control of growth and cross tolerance to stress. *Environ. Exp. Bot.* 94 73–88. 10.1016/j.envexpbot.2012.05.003

[B10] Benhar M. (2015). Nitric oxide and the thioredoxin system: a complex interplay in redox regulation. *Biochim. Biophys. Acta* 1850 2476–2484. 10.1016/j.bbagen.2015.09.010 26388496

[B11] Besson-Bard A. Gravot A. Richaud P. Auroy P. Duc C. Gaymard F. (2009). Nitric oxide contributes to cadmium toxicity in *Arabidopsis* by promoting cadmium accumulation in roots and by up-regulating genes related to iron uptake. *Plant Physiol.* 1494 1302–1315. 10.1104/pp.108.133348 19168643 PMC2649387

[B12] Bita C. E. Gerats T. (2013). Plant tolerance to high temperature in a changing environment: scientific fundamentals and production of heat stress-tolerant crops. *Front. Plant Sci.* 4:273 10.3389/fpls.2013.00273 PMC3728475 23914193

[B13] Bouchard J. N. Yamasaki H. (2008). Heat stress stimulates nitric oxide production in *Symbiodinium microadriaticum*: a possible linkage between nitric oxide and the coral bleachin phenomenon. *Plant Cell Physiol.* 2 287–294. 10.1093/pcp/pcn037 18308760

[B14] Camejo D. Romero-Puertas Mdel C. Rodríguez-Serrano M. Sandalio L. M. Lázarom J. J. Sevilla (2013). Salinity-induced changes in S-nitrosylation of pea mitochondrial proteins. *J. Proteomics* 79 87–99. 10.1016/j.jprot.2012.12.003 23238061

[B15] Cantrel C. Vazquez T. Puyaubert J. Reze N. Lesch M. Kaiser W. M. (2011). Nitric oxide participates in cold-responsive phosphophingolipid formation and gene expression in *Arabidopsis thaliana*. *New Phytol.* 189 415–427. 10.1111/j.1469-8137.2010.03500.x 21039566

[B16] Carvalho M. H. C. (2008). Drought stress and reactive oxygen species. *Plant Signal. Behav.* 3 156–165. 10.4161/psb.3.3.5536 19513210 PMC2634109

[B17] Chaki M. Shekariesfahlan A. Ageeva A. Mengel A. von Toerne C. Durner J. (2015). Identification of nuclear target proteins for S-nitrosylation in pathogen-treated *Arabidopsis thaliana* cell cultures. *Plant Sci.* 238 115–126. 10.1016/j.plantsci.2015.06.011 26259180

[B18] Chang R. Jang C. J. Branco-Price C. Nghiem P. Bailey-Serres J. (2012). Transient MPK6 activation in response to oxygen deprivation and reoxygenation is mediated by mitochondria and aids seedling survival in *Arabidopsis*. *Plant Mol. Biol.* 78 109–122. 10.1007/s11103-011-9850-5 22086331

[B19] Chaves M. M. Flexas J. Pinheiro C. (2009). Photosynthesis under drought and salt stress: regulation mechanisms from whole plant to cell. *Ann. Bot.* 103 551–560. 10.1093/aob/mcn125 18662937 PMC2707345

[B20] Cheng T. L. Chen J. H. Abd Allah E. F. Wang P. Wang G. P. Hu X. Y. (2015). Quantitative proteomics analysis reveals that S-nitrosoglutathione reductase (GSNOR) and nitric oxide signaling enhance poplar defense against chilling stress. *Planta* 242 1361–1390. 10.1007/s00425-015-2374-5 26232921

[B21] Chmielowska-Bak J. Gzyl J. Rucinska-Sobkowiak R. Arasimowicz-Jelonek M. Deckert J. (2014). The new insights into cadmium sensing. *Front. Plant Sci.* 5:245 10.3389/fpls.2014.00245 PMC4042028 24917871

[B22] Correa-Aragunde N. Foresi N. Lamattina L. (2015). Nitric oxide is an ubiquitous signal for maintaining redox balance in plant cells: regulation of ascorbate peroxidase as a case study. *J. Exp. Bot.* 66 2913–2921. 10.1093/jxb/erv073 25750426

[B23] Couturier J. Chibani K. Jacquot J. P. Rouhier N. (2013). Cysteine-based redox regulation and signaling in plants. *Front. Plant Sci.* 4:105 10.3389/fpls.2013.00105 PMC3638127 23641245

[B24] Cramer G. R. Urano K. Delrot S. Pezzotti M. Shinozaki K. (2011). Effects of abiotic stress on plants: a systems biology perspective. *BMC Plant Biol.* 11:163 10.1186/1471-2229-11-163 PMC3252258 22094046

[B25] Cui X. M. Zhang Y. K. Wu X. B. Liu C. S. (2010). The investigation of the alleviated effect of copper toxicity by exogenous nitric oxide in tomato plants. *Plant Soil Environ.* 56 274–281.

[B26] Das K. Roychoudhury A. (2014). Reactive oxygen species (ROS) and response of antioxidants as ROS-scavengers during environmental stress in plants. *Front. Environ. Sci.* 2:53 10.3389/fenvs.2014.00053

[B27] de Dios Barajas-López J. Blanco N. E. Strand Å (2013). Plastid-to-nucleus communication: signals controlling the running of the plant cell. *Biochim. Biophys. Acta* 1833 425–437. 10.1016/j.bbamcr.2012.06.020 22749883

[B28] De Michele R. Vurro E. Rigo C. Costa A. Elviri L. Di Valentin M. (2009). Nitric oxide is involved in cadmium-induced programmed cell death in *Arabidopsis* suspension cultures. *Plant Physiol.* 150 217–228. 10.1104/pp.108.133397 19261736 PMC2675725

[B29] de Montaigu A. Sanz-Luque E. Galván A. Fernández E. (2010). A soluble guanylate cyclase mediates negative signaling by ammonium on expression of nitrate reductase in *Chlamydomonas*. *Plant Cell* 22 1532–1548. 10.1105/tpc.108.062380 20442374 PMC2899865

[B30] del Río L. A. (2011). Peroxisomes as a source of reactive nitrogen species signal molecules. *Arch. Biochem. Biophys.* 506 1–11. 10.1016/j.abb.2010.10.022 21055384

[B31] del Río L. A. (2015). ROS and RNS in plant physiology: an overview. *J. Exp. Bot.* 66 2827–2837. 10.1093/jxb/erv099 25873662

[B32] del Río L. A. Sandalio L. M. Corpas F. J. Palma J. M. Barroso J. B. (2006). Reactive oxygen species and reactive nitrogen species in peroxisomes. Production, scavenging, and role in cell signaling. *Plant Physiol.* 141 330–335. 10.1104/pp.106.078204 16760483 PMC1475433

[B33] Demidchik V. (2015). Mechanisms of oxidative stress in plants: from classical chemistry to cell biology. *Environ. Exp. Bot.* 109 212–228. 10.1016/j.envexpbot.2014.06.021

[B34] Dietz K.-J. (2014). Redox regulation of transcription factors in plant stress acclimation and development. *Antioxid. Redox Signal.* 21 1356–1372. 10.1089/ars.2013.5672 24182193

[B35] Dodd A. N. Kudla J. Sanders D. (2010). The language of calcium signaling. *Annu. Rev. Plant Biol.* 61 593–620. 10.1146/annurev-arplant-070109-104628 20192754

[B36] Domingos P. Prado A. M. Wong A. Gehring C. Feijo J. A. (2015). Nitric oxide: a multitasked signaling gas in plants. *Mol. Plant* 8 506–520. 10.1016/j.molp.2014.12.010 25680232

[B37] Dordas C. (2015). “Nitric oxide and plant hemoglobins improve the tolerance of plant to hypoxia,” in *Nitric Oxide Action in Abiotic Stress Response to Plants* eds Khan M. N. Mobin M. Mohammad F. Corpas F. J. (Cham: Springer International Publishing). 10.1007/978-3-319-1784-2_1

[B38] Dubovskaya L. V. Bakakina Y. S. Kolesneva E. V. Sodel D. L. McAinsh M. R. Hetherington A. M. (2011). cGMP-dependent ABA-induced stomatal closure in the ABA-insensitive *Arabidopsis* mutant abi1-1. *New Phytol.* 191 57–69. 10.1111/j.1469-8137.2011.03661.x 21371039

[B39] Durner J. Wendehenne D. Klessig D. (1998). Defense gene induction in tobacco by nitric oxide, cyclic GMP, and cyclic ADP-Ribose. *Proc. Natl. Acad. Sci. U.S.A.* 95 10328–10333. 10.1073/pnas.95.17.10328 9707647 PMC21508

[B40] El-Maarouf-Bouteau H. Bailly C. (2008). Oxidative signaling in seed germination and dormancy. *Plant Signal. Behav.* 3 175–182. 10.4161/psb.3.3.5539 19513212 PMC2634111

[B41] Fan H. Li T. Guan L. Li Z. Guo N. Cai Y. (2012). Effects of exogenous nitric oxide on antioxidation and DNA methylation of *Dendrobium huoshanense* grown under drought stress. *Plant Cell Tissue Organ Cult.* 109 307–314. 10.1007/s11240-011-0096-3

[B42] Farnese F. S. Oliveira J. A. Gusman G. S. Leão G. A. Ribeiro C. Siman L. I. (2013). Plant responses to arsenic: the role of nitric oxide. *Water Air Soil Pollut.* 224 1660–1667. 10.1007/s11270-013-1660-8

[B43] Feigl G. Lehotia N. Molnár A. Ordog A. Rodríguez-Ruiz M. Palma J. M. (2015). Zinc induces distinct changes in the metabolism of reactive oxygen and nitrogen species (ROS and RNS) in the roots of two *Brassica* species with different sensitivity to zinc stress. *Ann. Bot.* 116 613–625. 10.1093/aob/mcu246 25538112 PMC4577986

[B44] Filippou P. Antoniou C. Fotopoulos V. (2013). The nitric oxide donor sodium nitroprusside regulates polyamine and proline metabolism in leaves of *Medicago truncatula* plants. *Free Radic. Biol. Med.* 56 172–183. 10.1016/j.freeradbiomed.2012.09.037 23041351

[B45] Filippou P. Bouchagier P. Skotti E. Fotopoulos V. (2014). Proline and reactive oxygen/nitrogen species metabolism is involved in the tolerant response of the invasive plant species *Ailanthus altissima* to drought and salinity. *Environ. Exp. Bot.* 97 1–10. 10.1016/j.envexpbot.2013.09.010

[B46] Floryszak-Wieczorek J. ArasimowiczJelonek M. Milczarek G. Janus L. Pawlak-Sprada S. Abramowski D. (2012). Nitric oxide-mediated stress imprint in potato as an effect of exposure to a priming agent. *Mol. Plant Microbe Interact.* 25 1469–1477. 10.1094/MPMI-02-12-0044-R 22835274

[B47] Foyer C. H. (2005). Redox homeostasis and antioxidant signaling: a metabolic interface between stress perception and physiological responses. *Plant Cell* 17 1866–1875. 10.1105/tpc.105.033589 15987996 PMC1167537

[B48] Francis S. H. Busch J. L. Corbin J. D. (2010). cGMP-dependent protein kinases and cGMP phosphodiesterases in nitric oxide and cGMP action. *Pharmacol. Rev.* 62 525–563. 10.1124/pr.110.002907 20716671 PMC2964902

[B49] Freschi L. Rodrigues M. A. Domingues D. S. Purgatto E. Van Sluys M. A. Magalhaes J. R. (2010). Nitric oxide mediates the hormonal control of crassulacean acid metabolism expression in young pineapple plants. *Plant Physiol.* 152 1971–1985. 10.1104/pp.109.151613 20147491 PMC2850025

[B50] Fungillo L. Skelly M. J. Loake G. J. Spoel S. H. Salgado I. (2014). S-nitrosothiols regulate nitric oxide production and storage in plants through the nitrogen assimilation pathway. *Nat. Commun.* 5 54001 10.1038/ncomms6401 PMC4229994 25384398

[B51] Gadjev I. Vanderauwera S. Gechev T. S. Laloi C. Minkov I. N. Shulaev V. (2006). Transcriptomic footprints disclose specificity of reactive oxygen species signaling in *Arabidopsis*. *Plant Physiol.* 141 436–445. 10.1104/pp.106.078717 16603662 PMC1475436

[B52] Gas E. Flores-Pérez U. Sauret-Güeto S. Rodríguez-Concepción M. (2009). Hunting for plant nitric oxide synthase provides new evidence of a central role for plastids in nitric oxide metabolism. *Plant Cell* 21 18–23. 10.1105/tpc.108.065243 19168714 PMC2648077

[B53] Gaupels F. Kuruthukulangarakoola G. T. Durner J. (2011). Upstream and downstream signals of nitric oxide in pathogen defence. *Curr. Opin. Plant Biol.* 14 707–714. 10.1016/j.pbi.2011.07.005 21816662

[B54] Gilroy S. Suzuki N. Miller G. Choi W.-G. Toyota M. Devireddy A. R. (2014). A tidal wave of signals: calcium and ROS at the forefront of rapid systemic signaling. *Trends Plant Sci.* 19 623–630. 10.1016/j.tplants.2014.06.013 25088679

[B55] Green J. Rolfe M. D. Smith L. J. (2014). Transcriptional regulation of bacterial virulence gene expression by molecular oxygen and nitric oxide. *Virulence* 5 794–809. 10.4161/viru.27794 25603427 PMC4601167

[B56] Groß F. Dumer J. Gaupels F. (2013). Nitric oxide, antioxidants and prooxidants in plant defence responses. *Front. Plant Sci.* 29:419 10.3389/fpls.2013.00419 PMC3812536 24198820

[B57] Gupta B. Huang B. (2014). Mechanism of salinity tolerance in plants: physiological, biochemical, and molecular characterization. *Int. J. Genomics* 2014 701596 10.1155/2014/701596 PMC3996477 24804192

[B58] Gupta K. J. Igamberdiev A. U. Manjunatha G. Segu S. Moran J. F. Neelawarne B. (2011). The emerging roles of nitric oxide (NO) in plant mithocondria. *Plant Sci.* 181 520–526. 10.1016/j.plantsci.2011.03.018 21893247

[B59] Gupta K. L. Igamberdiev A. U. (2015). “Compartmentalization of reactive oxygen species and nitric oxide production in plant cells: an overview,” in *Reactive Oxygen and Nitrogen Species Signaling and Communication in Plants* eds Gupta K. J. Igamberdiev A. U. (Cham: Springer International Publishing) 215–237. 10.1007/978-3-319-10079-1_11

[B60] Habibi G. (2014). “Hydrogen peroxide (H2O2) generation, scavenging and signaling in plants,” in *Oxidative Damage to Plants: Antioxidant Networks and Signalin* ed. Ahmad P. (San Diego, CA: Elsevier) 557–584. 10.1016/B978-0-12-799963-0.00019-8

[B61] Hasanuzzaman M. Fujita M. (2013). Exogenous sodium nitroprusside alleviates arsenic-induced oxidative stress in wheat (*Triticum aestivum* L.) seedlings by enhancing antioxidant defense and glyoxalase system. *Ecotoxicology* 22 584–596. 10.1007/s10646-013-1050-4 23430410

[B62] Hasanuzzaman M. Nahar K. Alam M. M. Roychowdhury R. Fujita M. (2013). Physiological, biochemical, and molecular mechanisms of heat stress tolerance in plants. *Int. J. Mol. Sci.* 14 9643–9684. 10.3390/ijms14059643 23644891 PMC3676804

[B63] Herrera-Vásquez A. Salinas P. Holuigue L. (2015). Salicylic acid and reactive oxygen species interplay in the transcriptional control of defense genes expression. *Front. Plant Sci.* 6:171 10.3389/fpls.2015.00171 PMC4365548 25852720

[B64] Hirabayashi Y. Mahendran R. Koirala S. Konoshima L. Yamazaki D. Watanabe S. (2013). Global flood risk under climate change. *Nat. Clim. Chang.* 3 819–821. 10.1038/nclimate1911

[B65] Horchani F. Prévot M. Boscari A. Evangelisti E. Meilhoc E. Bruand C. (2011). Both plant and bacterial nitrate reductases contribute to nitric oxide production in *Medicago truncatula* nitrogen-fixing nodules. *Plant Physiol.* 155 1023–1036. 10.1104/pp.110.166140 21139086 PMC3032450

[B66] Hossain M. A. Bhattacharjee S. Armin S. M. Qian P. Xin W. Li H. Y. (2015). Hydrogen peroxide priming modulates abiotic oxidative stress tolerance: insights from ROS detoxification and scavenging. *Front. Plant Sci.* 6:420 10.3389/fpls.2015.00420 PMC4468828 26136756

[B67] Hung S. H. Wang C. C. Ivanov S. V. Alexieva V. Yu C. W. (2007). Repetition of hydrogen peroxide treatment induces a chilling tolerance comparable to dold acclimation in mung bean. *J. Am. Soc. Hortic. Sci.* 132 770–776. 10.3389/fpls.2015.00420

[B68] Igamberdiev A. U. Hill R. D. (2004). Nitrate, NO and haemoglobin in plant adaptation to hypoxia: an alternative to classic fermentation pathways. *J. Exp. Bot.* 55 2473–2482. 10.1093/jxb/erh272 15448180

[B69] Íseri O. D. Körpe D. A. Sahin F. I. Haberal M. (2013). Hydrogen peroxide pretreatment of roots enhanced oxidative stress response of tomato under cold stress. *Acta Physiol. Plant.* 35 1905–1913. 10.1007/s11738-013-1228-7

[B70] Jajic I. Sarna T. Strzalka K. (2015). Senescence, stress, and reactive oxygen species. *Planta* 4 393–411. 10.3390/plants4030393 PMC4844410 27135335

[B71] Jasid S. Simontacchi M. Bartoli C. G. Puntarulo S. (2006). Chloroplasts as a nitric oxide cellular source: effect of reactive nitrogen species on chloroplastic lipids and proteins. *Plant Physiol.* 142 1246–1255. 10.1104/pp.106.086918 16980561 PMC1630751

[B72] Joo J. H. Bae Y. S. Lee J. S. (2001). Role of auxin-induced reactive oxygen species in root gravitropism. *Plant Physiol.* 126 1055–1060. 10.1104/pp.126.3.1055 11457956 PMC116462

[B73] Joudoi T. Shichiri Y. Kamizono N. Akaike T. Sawa T. Yoshitake J. (2013). Nitrated cyclic GMP modulates guard cell signaling in *Arabidopsis*. *Plant Cell* 25 558–571. 10.1105/tpc.112.105049 23396828 PMC3608778

[B74] Kneeshaw S. Gelineau S. Tada Y. Loake G. J. Spoel S. H. (2014). Selective protein denitrosylation activity of Thioredoxin-h5 modulates plant immunity. *Mol. Cell* 56 153–162. 10.1016/j.molcel.2014.08.003 25201412

[B75] Knight H. Trewavas A. J. Knight M. R. (1996). Cold calcium signaling in *Arabidopsis* involves two cellular pools and a change in calcium signature after acclimation. *Plant Cell* 8 489–503. 10.1105/tpc.8.3.489 8721751 PMC161115

[B76] Königshofer H. Tromballa H. W. Löppert H. G. (2008). Early events in signaling high-temperature stress in tobacco BY2 cells involve alterations in membrane flyidity and enhanced hydrogen peroxide production. *Plant Cell Environ.* 31 1771–1780. 10.1111/j.1365-3040.2008.01880.x 18761700

[B77] Kotvun Y. Chiu W.-L. Tena G. Sheen J. (2000). Functional analysis of oxidative stress-activated mitogen-activated protein kinase cascade in plants. *Proc. Natl. Acad. Sci. U.S.A.* 6 2940–2945. 10.1073/pnas.97.6.2940 PMC16034 10717008

[B78] Kumar S. Trivedi P. K. (2016). “Heavy metal stress signaling in plants,” in *Plant Metal Interaction – Emerging Remediation Techniques* ed. Ahmad P. (Amsterdan: Elsevier) 585–603. 10.1016/B978-0-12-803158-2.00025-4

[B79] Lamotte O. Bertoldo J. B. Besson-Bard A. Rosnoblet C. Aimé S. Hichami S. (2015). Protein S-nitrosylation: specificity and identification strategies in plants. *Front. Chem.* 2:114 10.3389/fchem.2014.00114 PMC4285867 25750911

[B80] Lázaro J. J. Jiménzez A. Camejo D. Iglesias-Baena I. Martí M. C. Lázaro-Payo A. (2013). Dissecting the integrative antioxidant and redox systems in plant mitochondria. Effect of stress and S-nitrosylation. *Front. Plant Sci.* 4:460 10.3389/fpls.2013.00460 PMC3842906 24348485

[B81] Leshem Y. Y. (2000). *Nitric Oxide in Plants. Occurrence, Function and Use*. Boston, MA: Kluwer Academic Publishers.

[B82] Leterrier M. Airaki M. Palma J. M. Chaki M. Barroso J. B. Corpas F. J. (2012). Arsenic triggers the nitric oxide (NO) and S-nitrosoglutathione (GSNO) metabolism in *Arabidopsis*. *Environ. Pollut.* 166 136–143. 10.1016/j.envpol.2012.03.012 22504427

[B83] Leterrier M. Chaki M. Airaki M. Valderrama R. Palma J. M. Barroso J. B. (2011). Function of S-nitrosoglutathione reductase (GSNOR) in plant development and under biotic/abiotic stress. *Plant Signal. Behav.* 6 789–793. 10.4161/psb.6.6.15161 21543898 PMC3218474

[B84] Li W. Liu X. Khan M. A. Yamaguchi S. (2005). The effect of plant growth regulators, nitric oxide, nitrate, nitrite and light on the germination of dimorphic seeds of *Suaeda* salsa under saline conditions. *J. Plant Res.* 118 207–214. 10.1007/s10265-005-0212-8 15937723

[B85] Lushchak V. I. (2015). Free radicals, reactive oxygen species, oxidative stress and its classification. *Chem. Biol. Interact.* 224 164–165. 10.1016/j.cbi.2014.10.016 25452175

[B86] Malik S. I. Hussain A. Yun B.-W. Spoel S. H. Loake G. J. (2011). GSNOR-mediated de-nitrosylation in the plant defence response. *Plant Sci.* 181 540–544. 10.1016/j.plantsci.2011.04.004 21893250

[B87] Marti M. C. Florez-Sarasa I. Camejo D. Pallol B. Ortiz A. RibasCarbo M. (2013). Response of mitochondrial antioxidant system and respiratory pathways to reactive nitrogen species in pea leaves. *Physiol. Plant.* 147 194–206. 10.1111/j.1399-3054.2012.01654.x 22607494

[B88] Mengel A. Chaki M. Shekariesfahlan A. Lidermayr C. (2013). Effect of nitric oxide on gene transcription – S-nitrosylation of nuclear proteins. *Front. Plant Sci.* 4:293 10.3389/fpls.2013.00293 PMC3729996 23914201

[B89] Millar T. M. Stevens C. R. Benjamin N. Eisenthal R. Harrison R. Blake D. R. (1998). Xanthine oxidoreductase catalyses the reduction of nitrates and nitrite to nitric oxide under hypoxic conditions. *FEBS Lett.* 8 225–228. 10.1016/S0014-5793(98)00430-X 9607316

[B90] Miller G. Suzuki N. Ciftci-Yilmaz S. Mittler R. (2010). Reactive oxygen species homeostasis and signalling during drought and salinity stresses. *Plant Cell Environ.* 33 453–467. 10.1111/j.1365-3040.2009.02041.x 19712065

[B91] Mittler R. Blumwald E. (2015). The roles of ROS and ABA in systemic acquired acclimation. *Plant Cell* 27 64–70. 10.1105/tpc.114.133090 25604442 PMC4330577

[B92] Modolo L. V. Augusto O. Almeida I. M. G. Magalhaes J. R. Salgado I. (2005). Nitrite as the major source of nitric oxide production by *Arabidopsis thaliana* in response to *Pseudomonas syringae*. *FEBS Lett.* 579 3814–3820. 10.1016/j.febslet.2005.05.078 15978583

[B93] Molassiotis A. Tanou G. Diamantidis G. (2010). NO says more than “Yes” to salt tolerance: salt priming and systemic nitric oxide signaling in plants. *Plant Signal. Behav.* 5 209–212. 10.4161/psb.5.3.10738 20061805 PMC2881262

[B94] Møller I. M. Sweetlove L. J. (2010). ROS signalling – specificity is required. *Trends Plant Sci.* 15 370–374. 10.1016/j.tplants.2010.04.008 20605736

[B95] Morales R. C. Bahnson E. S. M. Havelka G. E. Cantu-Medellin N. Kelley E. E. Kibbe M. R. (2015). Sex-based differential regulation of oxidative stress in the vasculature by nitric oxide. *Redox Biol.* 4 226–233. 10.1016/j.redox.2015.01.007 25617803 PMC4803798

[B96] Mulaudzi N. Ludidi O. Ruzvidzo M. Morse N. Hendricks E. Iwuoha C. (2011). Identification of a novel *Arabidopsis thaliana* nitric oxide-binding molecule with guanylate cyclase activity in vitro. *FEBS Lett.* 585 2693–2697. 10.1016/j.febslet.2011.07.023 21820435

[B97] Muns R. Tester M. (2008). Mechanisms of salinity tolerance. *Annu. Rev. Plant Biol.* 59 651–681. 10.1146/annurev.arplant.59.032607.092911 18444910

[B98] Mur L. A. J. Mandon J. Persijn S. Cristescu S. M. Moshkov I. E. Novikova G. V. (2013). Nitric oxide in plants: an assessment of the current state of knowledge. *AoB Plants* 5:pls052 10.1093/aobpla/pls052 PMC3560241 23372921

[B99] Mur L. A. J. Sivakumaran A. Mandon J. Cristescu S. M. Harren F. J. M. Hebelstrup K. H. (2012). Haemoglobin modulates salicylate and jasmonate/ethylene resistance mechanisms against pathogens. *J. Exp. Bot.* 63 4375–4387. 10.1093/jxb/ers116 22641422 PMC3421983

[B100] Neill S. Barros R. Bright J. Desikan R. Hancock J. Harrison J. (2008). Nitric oxide, stomatal closure, and abiotic stress. *J. Exp. Bot.* 59 165–176. 10.1093/jxb/erm293 18332225

[B101] Neill S. J. Desikan R. Clark A. Hurst R. D. Hancock J. T. (2002). Hydrogen peroxide and nitric oxide as signaling molecules in plants. *J. Exp. Bot.* 53 1237–1247. 10.1093/jexbot/53.372.1237 11997372

[B102] Neill S. J. Desikan R. Hancock J. T. (2003). Nitric oxide signalling in plants. *New Phytol.* 159 11–35. 10.1046/j.1469-8137.2003.00804.x 33873677

[B103] Nott A. Watson P. M. Robinson J. D. Crepaldi L. Riccio A. (2008). S-Nitrosylation of histone deacetylase 2 induces chromatin remodelling in neurons. *Nature* 18 411–415. 10.1038/nature07238 18754010

[B104] Opdenakker K. Remans T. Vangronsveld J. Cuypers A. (2012). Mitogen-activated protein (map) kinases in plant metal stress: regulation and responses in comparison to other biotic and abiotic stresses. *Int. J. Mol. Sci.* 13 7828–7853. 10.3390/ijms13067828 22837729 PMC3397561

[B105] Osakabe Y. Osakabe K. Shinozaki K. Tran L.-S. P. (2014). Response of plants to water stress. *Front. Plant Sci.* 5:86 10.3389/fpls.2014.00086 PMC3952189 24659993

[B106] Oz M. T. Eyidogan F. Yucel M. Öktem H. A. (2015). “Functional role of nitric oxide under abiotic stress conditions,” in *Nitric Oxide Action in Abiotic Stress Responses in Plants* eds Khan M. N. Mobin M. Mohammad F. Corpas F. J. (Cham: Springer International Publishing) 193–219. 10.1007/978-3-319-17804-2_2

[B107] Parry M. A. J. Andralojc P. J. Khan S. Lea P. J. Keys A. J. (2002). Rubisco activity: effects of drought stress. *Ann. Bot.* 89 833–839. 10.1093/aob/mcf103 12102509 PMC4233807

[B108] Pasqualini S. Meier S. Gehring C. Madeo L. Fornaciari M. Romano B. (2009). Ozone and nitric oxide induce cGMP-dependent and –independent transcription of defence genes in tobacco. *New Phytol.* 181 860–870. 10.1111/j.1469-8137.2008.02711.x 19140946

[B109] Pastori G. M. Kiddle G. Antoniw J. Bernard S. Veljovic-Jovanovic S. Verrier P. J. (2003). Leaf vitamin C contents modulate plant defense transcripts and regulate genes that control development through hormone signalling. *Plant Cell* 15 939–951. 10.1105/tpc.010538 12671089 PMC152340

[B110] Patel R. P. McAndrew J. Sellak H. White C. R. Jo H. Freeman B. A. (1999). Biological aspects of reactive nitrogen species. *Biochim. Biohys. Acta* 1411 385–400. 10.1016/s0005-2728(99)00028-6 10320671

[B111] Pei Z.-M. Murata Y. Benning G. Thomine S. Klusener B. Allen G. J. (2000). Calcium channels activated by hydrogen peroxide mediate abscisic acid signalling in guard cells. *Nature* 406 731–734. 10.1038/35021067 10963598

[B112] Perazzolli M. Dominici P. Romero-Puertas M. C. Zago E. Zeier J. Sonoda M. (2004). *Arabidopsis* nonsymbiotic hemoglobin AHB1 modulates nitric oxide bioactivity. *Plant Cell* 16 2785–2794. 10.1105/tpc.104.025379 15367716 PMC520971

[B113] Potters G. Pasternak T. P. Guisez Y. Palme K. J. Jansen M. A. K. (2007). Stress-induced morphogenic responses: growing out of trouble? *Trends Plant Sci.* 12 98–105. 10.1016/j.tplants.2007.01.004 17287141

[B114] Prasad T. K. Anderson M. D. Martin B. A. Stewart C. R. (1994). Evidence for chilling-induced oxidative stress in maize seedlings and a regulatory role for hydrogen-peroxide. *Plant Cell* 6 65–74. 10.2307/3869675 12244221 PMC160416

[B115] Puyaubert J. Fares A. Rézé N. Peltier J.-B. Baudouin E. (2014). Identification of endogenously S-nitrosylated proteins in *Arabidopsis* plantlets: effect of cold stress on cysteine nitrosylation level. *Plant Sci.* 21 150–156. 10.1016/j.plantsci.2013.10.014 24388526

[B116] Quiang J. Chen F. Kovalenkov Y. Pandey D. Moseley M. A. Foster M. W. (2012). Nitric oxide reduces NADPH oxidase 5 (Nox5) activity by reversible S-nitrosylation. *Free Radic. Biol. Med.* 52 1806–1819. 10.1016/j.freeradbiomed.2012.02.029 22387196 PMC3464050

[B117] Rahman T. Hosen I. Islam M. M. T. Shekhar H. U. (2012). Oxidative stress and human health. *Adv. Biosci. Biotechnol.* 3 997–1019. 10.4236/abb.2012.327123

[B118] Rentel M. C. Lecourieux D. Ouaked F. Usher S. L. Petersen L. Okamoto H. (2004). OXI1 kinase is necessary for oxidative burst-mediated signalling in *Arabidopsis*. *Nature* 427 858–861. 10.1038/nature02353 14985766

[B119] Rhoads D. M. Subbaiah C. C. (2007). Mitochondrial retrograde regulation in plants. *Mitochondrion* 7 177–194. 10.1016/j.mito.2007.01.002 17320492

[B120] Rodriguez M. C. S. Petersen M. Mundy J. (2010). Mitogen-activated protein kinase signalin in plants. *Annu. Rev. Plant Biol.* 61 621–649. 10.1146/annurev-arplant-042809-112252 20441529

[B121] Rümer S. Kapuganti J. G. Kaiser W. M. (2009). Oxidation of hydroxylamines to NO by plant cells. *Plant Signal. Behav.* 4 853–855. 10.1093/jxb/erp077 19847118 PMC2802794

[B122] Sandalio L. M. Romero-Puertas M. C. (2015). Peroxisomes sense and respond to environmental cues by regulating ROS and RNS signalling networks. *Ann. Bot.* 116 475–485. 10.1093/aob/mcv074 26070643 PMC4577995

[B123] Santisree P. Bhatnagar-Mathur P. Sharma K. K. (2015). NO to drought-multifunctional role of nitric oxide in plant drought: do we have all the answers? *Plant Sci.* 239 44–55. 10.1016/j.plantsci.2015.07.012 26398790

[B124] Sanz-Luque E. Ocana-Calahorro F. Montaigu A. Chamizo-Ampudia A. Llamas A. Galván A. (2015). THB1, a truncated hemoglobin, modulates nitric oxide levels and nitrate reductase activity. *Plant J.* 81 467–479. 10.1111/tpj.12744 25494936

[B125] Saxena I. Shekhawat G. S. (2013). Nitric oxide (NO) in alleviation of heavy metal induced phytotoxicity and its role in protein nitration. *Nitric Oxide* 32 13–20. 10.1016/j.niox.2013.03.004 23545403

[B126] Schulz P. Herde M. Romeis T. (2013). Calcium-dependent protein kinases: hubs in plant stress signaling and development. *Plant Physiol.* 163 523–530. 10.1104/pp.113.222539 24014579 PMC3793034

[B127] Seligman K. Saviani E. E. Oliveiram H. C. Pinto-Maglio C. A. F. Salgado I. (2008). Floral transition and nitric oxide emission during flower development in *Arabidopsis thaliana* is affected in nitrate reductase-deficient plants. *Plant Cell Physiol.* 49 1112–1121. 10.1093/pcp/pcn089 18540030

[B128] Sevilla F. Camejo D. Ortiz-Espín A. Calderón A. Lázaro J. J. Jiménez A. (2015). The thioredoxin/peroxiredoxin/sulfiredoxin system: current overview on its redox function in plants and regulation by reactive oxygen and nitrogen species. *J. Exp. Bot.* 66 2945–2955. 10.1093/jxb/erv146 25873657

[B129] Shao H.-B. Chu L. Shao M. Jaleel C. A. Hong-mei M. (2008). Higher plant antioxidants and redox signaling under environmental stresses. *C. R. Biol.* 331 433–441. 10.1016/j.crvi.2008.03.011 18510996

[B130] Shapiguzov A. Vainonen J. P. Wrzaczek M. Kangasjärvi J. (2012). ROS-talk – how the apoplast, the chloroplast, and the nucleus get the message through. *Front. Plant Sci.* 3:292 10.3389/fpls.2012.00292 PMC3530830 23293644

[B131] Shi H. Ye T. Zhu J.-K. Chan Z. (2014). Constitutive production of nitric oxide leads to enhanced drought stress resistance and extensive transcriptional reprogramming in *Arabidopsis*. *J. Exp. Bot.* 65 4119–4131. 10.1093/jxb/eru184 24868034 PMC4112625

[B132] Shi Y.-F. Wang D.-L. Wang C. Culler A. H. Kreiser M. A. Suresh J. (2015). Loss of GSNOR1 function leads to compromised auxin signaling and polar auxin transport. *Mol. Plant* 8 1350–1356. 10.1016/j.molp.2015.04.008 25917173

[B133] Sierla M. Rahikainen M. Salojärvi J. Kangasjärvi J. Kangasjärvi S. (2013). Apoplastic and chloroplastic redox signaling networks in plant stress responses. *Antioxid. Redox Signal.* 18 2220–2239. 10.1089/ars.2012.5016 23157163

[B134] Silveira N. M. Oliveira J. A. Ribeiro C. Canatto R. A. Siman L. Farnese F. (2015). Nitric oxide attenuates oxidative stress induced by arsenic in lettuce (*Lactuca sativa*) leaves. *Water Air Soil Pollut.* 226 379 10.1007/s11270-015-2630-0

[B135] Singh R. Gautam N. Mishra A. Gupta R. (2011). Heavy metals and living systems: an overview. *Indian J. Pharmacol.* 43 246–253. 10.4103/0253-7613.81505 21713085 PMC3113373

[B136] Song L. Ding W. Zhao M. Sun B. Zhang L. (2006). Nitric oxide protects against oxidative stress in the calluses from two ecotypes of reed. *Plant Sci.* 171 449–458. 10.1016/j.plantsci.2006.05.002 25193642

[B137] Steffens B. Kovalev A. Gorb S. N. Sauter M. (2012). Emerging roots alter epidermal cell fate through mechanical and reactive oxygen species signaling. *Plant Cell* 24 3296–3306. 10.1105/tpc.112.101790 22904148 PMC3462632

[B138] Stöhr C. Strube F. Marx G. Ullrich W. R. Rockel P. (2001). A plasma-membrane-bound enzyme of tobacco roots catalyzes the formation of nitric oxide from nitrite. *Planta* 212 835–841. 10.1007/s004250000447 11346959

[B139] Suzuki N. Mittler R. (2006). Reactive oxygen species and temperature stress: a delicate balance between signaling and destruction. *Physiol. Plant.* 126 45–51. 10.1007/s00709-010-0206-9

[B140] Tangahu B. V. Abdullah S. R. S. Basri H. Idris M. Anuar N. Mukhlisin M. (2011). A review on heavy metals (as, pb, and hg) uptake by plants through phytoremediation. *Int. J. Chem. Eng.* 2011 939161 10.1155/2011/939161

[B141] Tanou G. Job C. Rajjou L. Arc E. Belghazi M. Diamantidis G. (2009). Proteomics reveal the overlapping roles of hydrogen peroxide and nitric oxide in the acclimation of citrus plants to salinity. *Plant J.* 60 795–804. 10.1111/j.1365-313X.2009.04000 19682288

[B142] Tavares C. P. Vernal J. Delena R. A. Lamanttina L. Cassia R. Terenzi H. (2014). S-nitrosylation influences the structure and DNA binding activity of AtMYB30 transcription factor from *Arabidopsis thaliana*. *Biochim. Biophys. Acta* 1844 810–817. 10.1016/j.bbapap.2014.02.015 24583075

[B143] Terrile M. C. Paris R. Calderon-Villalobos L. I. Iglesias M. J. Lamattina L. Estelle M. (2012). Nitric oxide influences auxin signaling through S-nitrosylation of the *Arabidopsis* TRANSPORT INHIBITOR RESPONSE 1 auxin receptor. *Plant J.* 70 492–500. 10.1111/j.1365-313X.2011.04885.x 22171938 PMC3324642

[B144] Thao N. P. Khan M. I. R. Thu N. B. A. Hoang X. L. T. Asgher M. Khan N. (2015). Role of ethylene and its cross talk with other signaling molecules in plant responses to heavy metal stress. *Plant Physiol.* 169 73–84. 10.1104/pp.15.00663 26246451 PMC4577409

[B145] Thomas D. D. Ridnour L. A. Isenberg J. S. Flores-Santana W. Switzer C. H. Donzelli S. (2008). The chemical biology of nitric oxide: implications in cellular signaling. *Free Radic. Biol. Med.* 45 18–31. 10.1016/j.freeradbiomed.2008.03.020 18439435 PMC2572721

[B146] Tripathy B. C. Oelmüller R. (2012). Reactive oxygen species generation and signaling in plants. *Plant Signal. Behav.* 7 1621–1633. 10.4161/psb.22455 23072988 PMC3578903

[B147] Uchida A. Jagendorf A. T. Hibino T. Takabe T. (2002). Effects of hydrogen peroxide and nitric oxide on both salt and heat stress tolerance in rice. *Plant Sci.* 163 515–523. 10.1016/S0168-9452(02)00159-0

[B148] Valderrama R. Corpas F. J. Carreras A. Fernández-Ocanã A. Chaki M. Luque F. (2007). Nitrosative stress in plants. *FEBS Lett.* 581 453–461. 10.1016/j.febslet.2007.01.006 17240373

[B149] Verslues P. E. Bray E. A. (2006). Role of abscisic acid (ABA) and *Arabidopsis thaliana* ABA-insensitive loci in low water potential-induced ABA and proline accumulation. *J. Exp. Bot.* 57 201–212. 10.1093/jxb/erj026 16339784

[B150] Viehweger K. (2014). How plants cope with heavy metals. *Bot. Stud.* 55 1–12. 10.1186/1999-3110-55-35 PMC5432744 28510963

[B151] Voesenek L. A. C. J. Bailey-Serres J. (2015). Flood adaptative traits and process: and overview. *New Phytol.* 206 57–73. 10.1111/nph.13209 25580769

[B152] Wang D. Liu Y. Tan X. Liu H. Zeng G. Hu X. (2015). Effect of exogenous nitric oxide on antioxidative system and S-nitrosylation in leaves of *Boehmeria nivea* (L.) Gaud under cadmium stress. *Environ. Sci. Pollut. Res.* 22 3489–3497. 10.1007/s11356-014-3581-5 25242592

[B153] Wang H. Liang X. Wan Q. Wang X. Bi Y. (2009). Ethylene and nitric oxide are involved in maintaining ion homeostasis in *Arabidopsis thaliana* under salt stress. *Planta* 230 293–307. 10.1007/s00425-009-0946-y 19455351

[B154] Wang L. Su H. Han L. Wang C. Sun Y. Liu F. (2014). Differential expression profiles of poplar MAP kinase kinases in response to abiotic stresses and plant hormones, and overexpression of PtMKK4 improves the drought tolerance of poplar. *Gene* 545 141–148. 10.1016/j.gene.2014.04.058 24780863

[B155] Wang X. Hargrove M. S. (2013). Nitric oxide in plants: the roles of ascorbate and hemoglobin. *PLoS ONE* 8:e82611 10.1371/journal.pone.0082611 PMC3869716 24376554

[B156] Wang X. Hou C. Liu J. He W. Nan W. Gong H. (2013). Hydrogen peroxide is involved in the regulation of rice (*Oryza sativa* L.) tolerance to salt stress. *Acta Physiol. Plant.* 35 891–900. 10.1007/s11738-012-1132-6

[B157] Wang X. Zhang M. M. Wang Y. J. Gao Y. T. Li R. Wang G. F. (2015). The plasma membrane NADPH oxidase OsRbohA plays a crucial role in developmental regulation and drought-stress response in rice. *Physiol. Plant.* 24 1–34. 10.1111/ppl.12389 26400148

[B158] Wang Y. Li J. Wang J. Li Z. (2010). Hydrogen peroxide-mediated activity of map kinase 6 modulates nitric oxide biosynthsis and signal transduction in *Arabidopsis*. *Plant Cell* 22 2981–2998. 10.1105/tpc.109.072959 20870959 PMC2965546

[B159] Waszczak C. Akter S. Eeckhout D. Persiau G. Wahni K. Bodra N. (2014). Sulfenome mining in *Arabidopsis thaliana*. *Proc. Natl. Acad. Sci. U.S.A.* 111 11545–11550. 10.1073/pnas.1411607111 25049418 PMC4128149

[B160] Waszczak C. Akter S. Jacques S. Huang J. Messens J. Van Breusegem F. (2015). Oxidative post-translational modifications of cysteine residues in plant signal transduction. *J. Exp. Bot.* 66 2923–2934. 10.1093/jxb/erv084 25750423

[B161] Woodward A. W. Bartel B. (2005). Auxin: regulation, action, and interaction. *Ann. Bot.* 95 707–735. 10.1093/aob/mci083 15749753 PMC4246732

[B162] Wullf A. Oliveira H. C. Saviani E. E. Salgado I. (2009). Nitrite reduction and superoxide-dependent nitric oxide degradation by *Arabidopsis* mitochondria: influence of external NAD(P)H dehydrogenases and alternative oxidase in the control of nitric oxide levels. *Nitric Oxide* 21 132–139. 10.1016/j.niox.2009.06.003 19576290

[B163] Xia X.-J. Zhou Y.-H. Shi K. Zhou J. Foyer C. H. Yu J.-Q. (2015). Interplay between reactive oxygen species and hormones in the control of plant development and stress tolerance. *J. Exp. Bot.* 66 2839–2856. 10.1093/jxb/erv089 25788732

[B164] Xu H. Sun X. Wang X. Shi Q. Yang X. Yang F. (2011). Involvement of a cucumber MAPK gene (CsNMAPK) in positive regulation of ROS scavengence and osmotic adjustment under salt stress. *Sci. Hortic.* 127 488–493. 10.1016/j.scienta.2010.11.013

[B165] Yadav S. David A. Baluska F. Bhatla S. C. (2013). Rapid auxin-induced nitric oxide accumulation and subsequent tyrosine nitration of proteins during adventitious root formation in sunflower hypocotyls. *Plant Signal. Behav.* 8:e23196 10.4161/psb.23196 PMC3676489 23299324

[B166] Yang L. Fountain J. C. Wang H. Ni X. Ji P. Lee R. D. (2015). Stress sensitivity is associated with differential accumulation of reactive oxygen and nitrogen species in maize genotypes with contrasting levels of drought tolerance. *Int. J. Mol. Sci.* 16 24791–24819. 10.3390/ijms161024791 26492235 PMC4632777

[B167] Yu C. W. Murphy T. M. Lin C. H. (2003). Hydrogen peroxide-induces chilling tolerance in mung beans mediated throught ABA-independent glutathione accumulation. *Funct. Plant Biol.* 30 955–963. 10.1071/FP03091 32689080

[B168] Yu M. Lamattina L. Spoel S. H. Loake G. J. (2014). Nitric oxide function in plant biology: a redox cue in deconvolution. *New Phytol.* 202 1142–1156. 10.1111/nph.12739 24611485

[B169] Yu Q. Sun L. Jin H. Chen Q. Chen Z. Xu M. (2012). Lead-induced nitric oxide generation plays a critical role in lead uptake by *Pogonatherum crinitum* root cells. *Plant Cell Phisiol.* 53 1728–1736. 10.1093/pcp/pcs116 22904111

[B170] Yun B. W. Feechan A. Yin M. Saidi B. B. N. Bihan T. L. Manda Y. (2011). S-nitrosylation of NADPH oxidase regulates cell death in plant immunity. *Nature* 478 264–268. 10.1038/nature10427 21964330

[B171] Zagorchev L. Seal C. E. Kranner I. Odjakova M. (2013). A central role for thiols in plant tolerance to abiotic stress. *Int. J. Mol. Sci.* 14 7405–7432. 10.3390/ijms14047405 23549272 PMC3645693

[B172] Zhang J. Z. Creelman R. A. Zhu J. K. (2004). From laboratory to field. Using information from *Arabidopsis* to engineer salt, cold and drought tolerance in crops. *Plant Physiol.* 135 615–621. 10.1104/pp.104.040295 15173567 PMC514097

[B173] Zhang Y. Wang L. Liu Y. Zhang Q. Wei Q. Zhang W. (2006). Nitric oxide enhances salt tolerance in maize seedlings through increasing activities of proton-pump and Na+/H+ antiport in the tonoplast. *Planta* 224 545–555. 10.1007/s00425-006-0242-z 16501990

[B174] Zhao L. Zhang F. Guo J. Yang Y. Li B. Zhang L. (2004). Nitric oxide functions as a signal in salt resistance in the calluses from two ecotypes of reed. *Plant physiol.* 134 849–857. 10.1104/pp.103.030023 14739346 PMC344559

[B175] Zhao M.-G. Chen L. Zhang L. Zhang W. (2009). Nitric reductase-dependent nitric oxide production is involved in cold acclimation and freezing tolerance in *Arabidopsis*. *Plant Physiol.* 151 755–767. 10.1104/pp.109.140996 19710235 PMC2754647

[B176] Zhao M.-G. Tian Q.-Y. Zhang W.-H. (2007). Nitric oxide synthase-dependent nitric oxide production is associated with salt tolerance in *Arabidopsis*. *Plant Physiol.* 144 206–217. 10.1104/pp.107.096842 17351048 PMC1913813

[B177] Zhou J. Wang J. Shi K. Xia X. J. Zhou Y. H. Yu J. Q. (2012). Hydrogen peroxide is involved in the cold acclimation-induced chilling tolerance of tomato plants. *Plant Physiol. Biochem.* 60 141–149. 10.1016/j.plaphy.2012.07.010 22935478

[B178] Zhu J. K. (2003). Regulation of ion homeostasis under salt stress. *Curr. Opin. Plant Biol.* 6 441–445. 10.1016/S1369-5266(03)00085-2 12972044

